# Efficacy of ETVAX, a vaccine against enterotoxigenic *Escherichia coli*-positive diarrhoea in Gambian children: a double-blind, randomised, placebo-controlled, phase 2b trial

**DOI:** 10.1016/S1473-3099(25)00774-1

**Published:** 2026-06

**Authors:** M Jahangir Hossain, Fatou Secka, Lady C Sanyang, Raifu Taiwo, Emmanuel C Okoh, Olubunmi A Olubiyi, Mbemba Drammeh, Emmanuel U Richard, Ahmed D Balami, Mama Drammeh, Samba Juma Jallow, Bakary Sonko, Paticia Ezedimbu-Michael, Jacinta Obiaduo, Ousman Secka, Joanna Kaim, Björn Sjöstrand, Agneta Lissmats, Nils Carlin, Umberto D'Alessandro, Ann-Mari Svennerholm, Thomas F Wierzba

**Affiliations:** a Medical Research Council Unit The Gambia at the London School of Hygiene & Tropical Medicine (MRCG at LSHTM), Banjul, The Gambia; b Scandinavian Biopharma, Solna, Sweden; c Department of Microbiology and Immunology, University of Gothenburg, Gothenburg, Sweden; d Section on Infectious Diseases, Wake Forest School of Medicine, Winston Salem, NC, USA

## Abstract

**Background:**

Enterotoxigenic *Escherichia coli* (ETEC) causes 75 million diarrhoea episodes with up to 42 000 deaths annually in children. To prevent ETEC in children, we aimed to evaluate the safety, immunogenicity, and efficacy of ETVAX, an oral, inactivated, whole-cell ETEC vaccine with toxoid and double-mutant heat-labile toxin adjuvant.

**Methods:**

In this phase 2b, double-blind, placebo-controlled trial, Gambian children aged 6–18 months were recruited from four enrolment centres and block-randomised (1:1) via a computer-generated sequence, stratified by enrolment centres, to receive ETVAX or placebo on days 1, 15, and 90. Parents, staff, investigators assessing outcomes, and investigators analysing the data were masked to group assignment. An immunogenicity subset was assessed for serum antibody responses to ETEC colonisation factors and heat-labile toxin. The primary safety endpoint was serious adverse events, assessed in all children who received at least one intervention dose. The primary efficacy endpoint was vaccine efficacy against moderate-to-severe ETEC-positive diarrhoea (MSD-ETEC), excluding co-infections with *Cryptosporidium* spp, norovirus genogroup II, rotavirus, or *Shigella* spp, assessed in the per-protocol population. Secondary endpoints included vaccine efficacy against MSD-ETEC regardless of copathogens and against moderate-to-severe diarrhoea (MSD) regardless of cause. Exploratory vaccine efficacy analyses were against MSD-ETEC excluding only enteroparasitic copathogens (*Giardia lamblia* and *Cryptosporidium*) and against MSD-ETEC regardless of copathogens when first dose was given before age 9 months. This trial was registered with the Pan African Clinical Trials Registry (PACTR20201081921856).

**Findings:**

Between Feb 22, 2021, and June 24, 2022, 5253 children were screened and 4936 (2499 [51%] girls, 2437 [49%] boys) were randomly assigned (2468 to ETVAX, 2468 to placebo). Serious adverse events occurred in 24 (1·0%) of 2474 in the vaccine group and 32 (1·3%) of 2462 in the placebo group, with none related to the investigational product. Immunogenicity was assessed in 122 children. ETVAX increased antibodies to colonisation factors (CFA/I, CS3) and heat-labile toxins. Vaccine efficacy was 26·6% (95% CI −58·3 to 66·0; p=0·43) for the primary endpoint, 48·2% (p=0·053) against MSD-ETEC regardless of copathogens, and 80·6% (p=0·0092) when excluding enteroparasitic copathogens. Vaccine efficacy against all MSD-ETEC reached 67·8% (p=0·026) when dosing started before age 9 months. Vaccine efficacy against MSD regardless of cause was 21·4% (p=0·032).

**Interpretation:**

ETVAX was safe and immunogenic. Although the primary endpoint was not met, the secondary and exploratory findings suggest ETVAX protects Gambian children against ETEC-positive MSD particularly when administered before age 9 months and when children were not co-infected with enteroparasites. ETVAX also showed a reduction in all-cause MSD. These results support advancing ETVAX to a pivotal phase 3 trial.

**Funding:**

European & Developing Countries Clinical Trials Partnership.

## Introduction

Annually, enterotoxigenic *Escherichia coli* (ETEC) causes 220 million diarrhoea episodes globally, with 75 million episodes and up to 42 000 deaths in children younger than 5 years mainly occurring in low-income countries.^1^ Climate models predict increased ETEC incidence under warming conditions.^2^ An ETEC vaccine could reduce illness and deaths, improve child growth, decrease health-care costs, and curb antimicrobial resistance.^3^, ^4^

ETEC causes diarrhoea by expressing proteinaceous colonisation factors, enabling bacterial adhesion to small intestine enterocytes. Among 26 distinct colonisation factors, CFA/I, CS3, CS5, and CS6 are the most common.^5^ After colonisation, ETEC release heat-labile toxins, heat-stable toxins, or both (LTST), which can result in severe diarrhoea and dehydration.^6^

**Research in context**

**Evidence before this study**

ETVAX is an oral whole-cell vaccine for enterotoxigenic *Escherichia coli* (ETEC) consisting of inactivated *E coli* bacteria overexpressing the most prevalent colonisation factors in combination with a toxoid (LCTBA) and the double-mutant heat-labile toxin (dmLT) adjuvant. ETVAX is designed to prevent bacterial colonisation and neutralisation of heat-labile toxin. Among ETEC vaccines in development, WHO considers ETVAX the leading candidate. We searched PubMed without language restrictions using the term “ETVAX” for publications from database inception to Nov 30, 2025, and identified five published clinical trials assessing Swedish, Zambian, Bangladeshi, and Finnish adults, as well as age-descending, dose-escalation studies in Bangladeshi and Zambian infants and children. Across these studies, ETVAX was consistently showed to be safe and immunogenic in all age groups. In the Zambian trial, a three-dose schedule using one-quarter of the adult dose with 2·5 μg dmLT was identified as the optimal vaccination regimen for infants and young children.

**Added value of this study**

This trial, conducted in nearly 5000 Gambian children aged 6–18 months, is the first study to evaluate the safety, immunogenicity, and efficacy of ETVAX in a large paediatric population in a low-income country. Using active and passive surveillance, we confirmed that ETVAX is safe and induces immune responses to colonisation factors and heat-labile toxins. Importantly, this study provides the first evidence that ETVAX can significantly reduce the incidence of ETEC-positive and all-cause diarrhoea, particularly when vaccination is initiated before age 9 months and in children without concurrent enteroparasitic infections.

**Implications of all the available evidence**

Multiple clinical trials have now shown consistent safety and immunogenicity of ETVAX across multiple geographical settings and age groups. This study provides the first demonstration of induction of protective efficacy by ETVAX in young children who are at risk. These findings support progression to a large, multicountry, phase 3 trial to confirm ETVAX efficacy against ETEC disease in children and to support ETVAX introduction in high-burden settings.

ETVAX is an oral vaccine designed to reduce ETEC-positive diarrhoea by inducing mucosal immunity to common ETEC colonisation factors and heat-labile toxins. At the vaccine's consultation in June, 2020, WHO's Product Development for Vaccines Advisory Committee described ETVAX as the leading ETEC vaccine candidate.^7^ We present findings from a large, phase 2b trial evaluating safety, immunogenicity, and efficacy of ETVAX in children in The Gambia.

## Methods

### Study design and participants

We conducted a double-blind, randomised, placebo-controlled, phase 2b trial in Gambian children living in rural and peri-urban communities. These areas, located along the River Gambia, are characterised by small-scale farming, dispersed settlements, and a subtropical climate with a single rainy season (June–October). The catchment area included 341 villages (population 188 066, with 18 139 children younger than 5 years), served by ten surveillance clinics, all located within government health and medical centres, four of which were enrolment centres for this study. Potentially eligible children were identified in villages and, if their parents were interested, transported by project vehicles to an enrolment centre for informed consent. Before enrolment, the aims, procedures, risks, and benefits of the trial were discussed with community leaders, families, and social groups.

Inclusion criteria were healthy children aged 6–18 months whose parents consented and were available for follow-up. Exclusion criteria were systemic or congenital disorders, febrile illness within the past 48 h, acute disease within the past 3 days, diarrhoea within the past 7 days, vaccination or medication use within the past 7 days (ie, antibiotics, iron or zinc supplements, or antacids), immunosuppressant use for more than 14 days, previous cholera vaccination, previous blood transfusion, and malnutrition (weight-for-length Z score lower than –2·0). The child's ethnicity and sex were reported by parents. Routine vaccine use was documented by reviewing the Gambian Ministry of Health child health cards.

The trial complied with the principles of the Declaration of Helsinki and the International Conference on Harmonisation of Good Clinical Practice guidelines. Ethical approval was obtained from The Gambia Government/Medical Research Council (MRC) Joint Ethics Committee and the London School of Hygiene & Tropical Medicine ethics committee. Written informed consent was obtained from parents for all children. The protocol, statistical analysis plan, and data are available upon reasonable request from Scandinavian Biopharma. The trial was registered with the Pan African Clinical Trials Registry (PACTR20201081921856).

### Randomisation and masking

Children were randomly assigned (1:1) via a computer-generated sequence in blocks of six stratified by enrolment centre to receive either vaccine (ETVAX) or placebo. The unmasked team generated the allocation sequence, accessed randomisation lists, and prepared the doses and had no other role in the trial. Parents of the children, staff administering the interventions, investigators assessing outcomes, and investigators analysing the data were masked to group assignment. Placebo doses matched the vaccine doses in appearance and volume to ensure masking.

### Procedures

Participants received three doses of the vaccine or placebo administered on days 1, 15, and 90 at the serveillance clinics. ETVAX is an oral, inactivated vaccine composed of four formaldehyde-inactivated or phenol-inactivated *E coli* strains, each genetically engineered to overexpress one colonisation factor: CFA/I, CS3, CS5, or CS6.^8^ Three of the four strains (CFA/I, CS3, and CS5) express O78 lipopolysaccharide; the CS6-expressing strain does not have O antigen. The vaccine includes the toxoid LCTBA,^9^ a chimera of the binding B subunits of heat-labile toxin and cholera toxin and is adjuvanted with double-mutant heat-labile toxin (dmLT).^10^ dmLT has been shown to be safe and well tolerated in children when administered with ETVAX.^11^ Each vaccine dose combines 10 mL sodium bicarbonate buffer, 2 × 10^10^ inactivated *E coli* (0·245 μg CFA/I, 0·783 μg CS3, 0·158 μg CS5, and 0·036 μg CS6), 250 μg LCTBA, and 2·5 μg dmLT. The placebo contained sodium bicarbonate buffer alone. Children fasted 60 min before and 30 min after dosing.

The inactivated *E coli* and LCTBA were produced and released by Cobra Biopharma Matfors (Matfors, Sweden) and the dmLT by IDT Biologika (Dessau-Rosslau, Germany). The sodium bicarbonate buffer powder was manufactured by Recipharm Höganäs (Höganäs, Sweden).

The first 350 enrolled children (ie, the reactogenicity cohort) underwent active home surveillance by trial nurses for adverse events for 7 days after vaccination using a checklist to assess vital signs and predefined symptoms (vomiting, diarrhoea or gastroenteritis, pyrexia, upper respiratory infection, urticaria, and allergic dermatitis). All children were monitored passively for unsolicited adverse events when seeking medical care via a form based on a predefined list of symptoms. Severity was graded by research clinicians with the US Division of AIDS criteria.^12^ Serious adverse events were defined as death, life-threatening conditions, hospitalisation, or persistent disability or incapacity. All events were classified by research clinicians as either related or unrelated to the investigational product with the WHO–Uppsala Monitoring Centre system.^13^ Trial physicians and nurses provided care for adverse events, with severe or life-threatening events managed by hospital specialists.

Diarrhoea surveillance occurred at the ten trial clinics, which included the four enrolment centres. Diarrhoea was defined as four or more loose or liquid stools within 24 h (amended on May 22, 2022, to three or more stools to align with global definitions)^14^, ^15^ occurring within 10 days after ≥3 consecutive diarrhoea-free days, distinguishing acute from prolonged or chronic events. Diarrhoea severity was measured based on the WHO dehydration treatment schedule and modified Vesikari score.^15^, ^16^ Moderate diarrhoea included one of the following: vomiting one or two times per 24 h, thirst, skin turgor of ≤2 s, or restlessness or irritability. Severe diarrhoea included one of the following: intravenous fluid use, hospitalisation, inability to drink, vomiting three or more times per 24 h, sunken eyes, lethargy, or skin turgor of >2 s.

Stool and rectal swabs were collected from children with moderate-to-severe diarrhoea (MSD). Rectal swabs were tested for ETEC toxin genes (heat-labile toxins, heat-stable toxins [human], and heat-stable toxins [porcine]) with the Novodiag Bacterial GE molecular assay (Hologic, Marlborough, MA, USA).^17^ If ETEC was detected, corresponding stool samples were sent to the MRC Gambia (MRCG) laboratory in Keneba for storage at −70°C and cultured for enteric pathogens. ETEC-positive isolates underwent PCR, dot blot, and GM1-ELISA assays as described.^18^ Confirmed isolates were shipped to Gothenburg, Sweden for quality control of colonisation factors and toxin expression.^18^, ^19^, ^20^ Additional PCR screening for other pathogens was performed at Synlab (Helsinki, Finland). *Campylobacter* spp were detected by ELISA at MRCG Fajara. For details see the appendix (pp 2–3)

### Outcomes

The primary safety endpoint was the incidence of serious adverse events. The primary efficacy endpoint was the first episode of clinically significant (ie, MSD) acute diarrhoea associated with culture-detected ETEC expressing heat-labile toxins, LTST, or heat-stable toxins with at least one colonisation factor (CFA/I, CS3, CS5, CS6) or without listed copathogens detected, from 7 days after receiving the third dose. Listed copathogens were *Cryptosporidium* spp, norovirus genogroup II (GII), rotavirus, or *Shigella* spp. These pathogens were excluded based on previous epidemiological data from The Gambia^21^ and other sub-Saharan countries^22^, ^23^, ^24^, ^25^ suggesting that these infections might have a greater probability of inducing diarrhoea than ETEC, and their inclusion could misclassify aetiology, masking ETEC-specific efficacy. In mixed ETEC infections (ie, two or more ETEC strains), at least one ETEC strain had to meet the toxin and colonisation factor criteria for ETEC-positive MSD.

Secondary endpoints used the same definition as the primary efficacy endpoint (first culture-confirmed ETEC acute MSD expressing heat-labile toxins, heat-stable toxins, or LTST with at least one of CFA/1, CS3, CS5, or CS6), but evaluated vaccine efficacy for ETEC-positive MSD regardless of copathogens and for MSD regardless of aetiology (all-cause diarrhoea). Exploratory analyses assessed ETEC-positive MSD after excluding those with enteroparasitic copathogens. A post-hoc analysis, based on an observed elevated incidence in the youngest Gambian children, evaluated efficacy by age at first dose (ie, first dose at 6 months to <9 months *vs* 9–18 months) for ETEC-positive and all-cause MSD.

Because the surveillance case definition for MSD was modified from at least four to at least three loose or liquid stools within 24 h, the definition was also changed for the primary endpoint and post-hoc analysis while maintaining all other clinical criteria.

### Statistical analysis

Extrapolating from Gambian data,^21^ we assumed an incidence of 1·87% for ETEC-positive MSD over 18 months among unvaccinated children. To detect 60% efficacy (0·75% incidence in the ETVAX group), we accounted for 85% coverage, 12% dropout, and 30% not seeking health care for MSD. With a one-sided p value of 0·05 and 80% power, based on a χ^2^ statistic, the number of participants per group was 2468, or 4936 children in total.

The safety population included all children who received at least one dose of the intervention. The intention-to-treat (ITT) population included children who were randomly assigned, regardless of dosing errors. The modified ITT (mITT) population included children who received three doses, analysed according to their randomised assignment. The per-protocol population included children receiving three doses without protocol deviations affecting efficacy endpoints. Exclusion decisions occurred before database lock.

For the primary safety analysis, the proportion of children who had solicited (reactogenicity cohort) and unsolicited adverse events (safety cohort) were summarised by type, severity, and treatment group. Solicited events were additionally summarised after each vaccine dose by treatment group. Crude efficacy was calculated as (1 – relative risk) × 100. Adjusted efficacy was (1 – hazard ratio) × 100. The hazard was obtained from multivariable Cox's proportional hazards model. Cox's model assumptions were assessed with methods described by Lin and colleagues;^26^ when assumptions were not met, models were stratified for non-proportionality. Variables significantly associated (p<0·10) in bivariate analysis were included in multivariable models, with final selection by backward elimination (p<0·05). Candidate variables were child's sex, child's age group at enrolment (<12 months *vs* 12 months or older), length-for-age Z score, mother's education (no formal schooling, less than primary, completed primary, or higher education), access to an improved water source, and access to an improved waste facility. Improved water and sanitation followed UNICEF standards.^27^ The enrolment centre was included regardless of significance (as a standard procedure) as randomisation was stratified by centre. Two-tailed p values and 95% CIs are presented. Withdrawn participants were right censored. Episodes occurring less than 7 days after the third dose were right censored at day 0. For the primary efficacy endpoint, if the first episode of ETEC expressing heat-labile toxins, heat-stable toxins, or LTST with CFA/I, CS3, CS5, or CS6 occurred with copathogens, the participant was right censored at the time of the episode. If the first episode of ETEC was not expressing heat-labile enterotoxin, but only heat-stable enterotoxin with no colonisation factors, the participant was right censored at the time of the episode. Additional efficacy endpoints followed similar rules depending on endpoint definition. A cumulative incidence curve was generated to display the probability of experiencing all-cause MSD over time by treatment group. The number of events and children at risk over time are given. Because the time-to-event analysis used right censoring for study withdrawals, no imputation for missing data was performed. Relative risk, vaccine efficacy, 95% CIs, and two-tailed p values were also calculated by age at first dose: 6 months to <9 months *vs* 9–18 months.

ETVAX targets a subset of ETEC strains, specifically those expressing heat-labile toxins, LTST, CFA/I, CS3, CS5, or CS6. To estimate coverage of local circulating strains, we calculated the proportion of ETEC-positive MSD events with isolates expressing at least one of these antigens. Coverage (%)=[(total ETEC strains regardless of toxin or colonisation factor expression – number of ETEC strains not expressing any ETVAX antigen) ÷ total ETEC strains regardless of toxin or colonisation factor expression] × 100. To reduce bias from vaccine-induced suppression of circulating strains among ETVAX recipients, the analysis was restricted to the placebo group. All ETEC strains from MSD events occurring after the first dose until the end of follow-up in the ITT population were included to improve precision.

The number needed to vaccinate (NNV) to prevent one MSD event is 1 divided by absolute risk reduction (ie, the difference in the incidence of diarrhoea between placebo and ETVAX recipients). The diarrhoea incidence was the number of events divided by the total number of children in that group. The NNV was reported for efficacy results with p<0·10.

For vaccine immunogenicity analyses, investigators aimed to select 150 children. This sample size was based on the feasibility of conducting large-scale blood draws given parental hesitancy to participate in venous blood collection. The immunogenicity cohort included all randomly assigned participants who received at least two doses of the vaccine or placebo and with at least two blood samples (one before vaccination). Children were selected, stratified by early enrolment (vaccinated by June 30, 2021) and late enrolment (vaccinated from Feb 1, 2022), to evaluate the consistency of vaccine immunogenicity over time. Of the 150 children, 75 were selected from the first 750 enrolled and 75 were selected from the last 750 enrolled (specifically every tenth child). Blood samples were collected on day 1 (before vaccination) and 7 days after the second and third doses. Serum IgA antibody titres against CFAs and heat-labile toxin B subunit (LTB) and IgG titres against LTB were measured by ELISA.^28^, ^29^ The immunogenicity analysis measured geometric mean titres (GMTs) of IgA antibodies against CFA/I, CS3, CS5, CS6, and LTB, and IgG antibodies against LTB before dosing and after the second and third doses. Geometric mean fold rises (GMFRs; ie, titre after immunisation divided by titre before immunisation) were compared with the Student's *t* test. The proportions of participants with two-fold or higher and four-fold or higher increases were also calculated.

Time-stratified sampling assessed vaccine-induced antibody responses by enrolment period: early enrolees (vaccinated by June 30, 2021) and late enrolees (vaccinated from Feb 1, 2022). GMFRs after two and three doses were compared between groups with the Student's *t* test.

No adjustments were made for multiple comparisons, and the results should be considered descriptive and hypothesis generating. An independent data safety and monitoring board regularly reviewed safety data. SAS version 9.4 was used for analyses.

### Role of the funding source

The funder had no role in study design, data collection, data analysis, data interpretation, or writing of the report.

## Results

Recruitment occurred from Feb 22, 2021, to June 24, 2022, with follow-up completed by Oct 31, 2023. 5253 children were screened for eligibility, resulting in the randomisation of 4936 children (2468 children per group), forming the ITT populations. Vaccine allocation errors yielded a safety cohort of 2474 children in the vaccine group and 2462 in the placebo group. The mITT population included 2184 participants in the vaccine group and 2177 in the placebo group; the per-protocol population included 2041 in the vaccine group and 2082 in the placebo group (figure). The reactogenicity cohort comprised 175 participants per group and immunogenicity was assessed in 122 children. The immunogenicity analysis included fewer children than planned due to parental hesitancy for child blood draws. In the vaccine group, there were 69 blood draws before dose 1, 63 after dose 2, and 46 after dose 3. In the placebo group, there were 53 blood draws before dose 1, 46 after dose 2, and 46 after dose 3. Among vaccine recipients, 34 early enrolees and 29 (or 26 depending on the antigen tested) late enrolees were evaluated.

**Figure:**
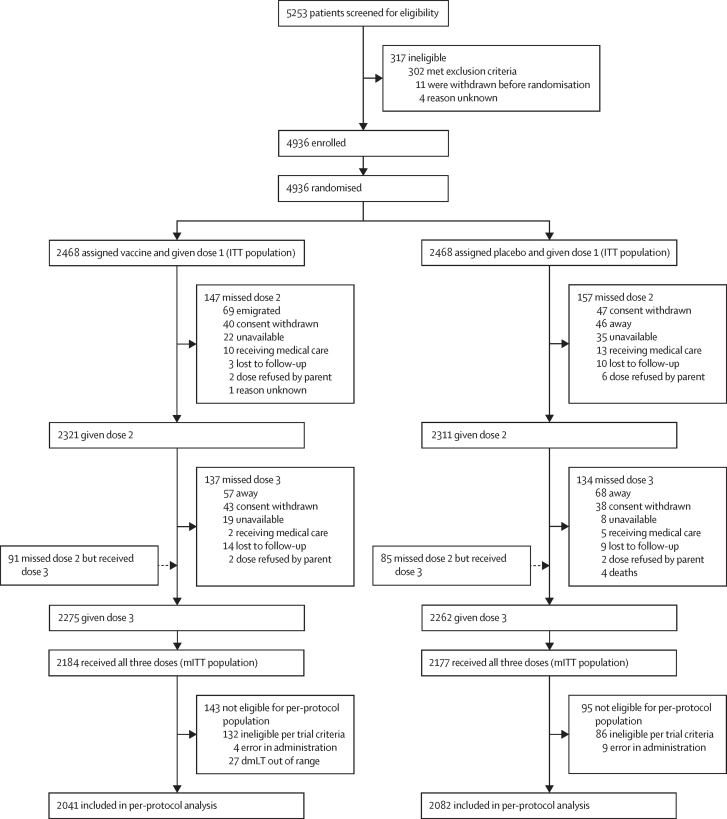
Trial profile dmLT=double-mutant heat-labile toxin. ITT=intention to treat. mITT=modified ITT.

Baseline demographic and clinical characteristics were similar between groups (table 1). Nearly all participants had received two rotavirus vaccine doses (Rotarix, GlaxoSmithKline) before enrolment.

**Table 1:** Characteristics of the intention-to-treat population by investigational product

	**Vaccine (n=2468)**	**Placebo (n=2468)**
**Child's sex**		
Girls	1277 (52%)	1222 (50%)
Boys	1191 (48%)	1246 (50%)
**Child's age, months**		
6 to <9	849 (34%)	826 (33%)
9 to <12	544 (22%)	570 (23%)
12 to 18	1075 (44%)	1072 (43%)
**Child's ethnic group**		
Mandinka	900 (36%)	919 (37%)
Wolof	762 (31%)	778 (32%)
Fula	617 (25%)	580 (24%)
Bambara	115 (5%)	120 (5%)
Other	71 (3%)	64 (3%)
Not reported or missing	3 (<1%)	7 (<1%)
**Child's measurements**		
Mean length, cm	71·8 (4·9)	72·1 (5·1)
Mean weight, kg	8·30 (1·19)	8·32 (1·21)
Mean weight for length, Z score	−0·42 (0·96)	−0·42 (0·97)
**Enrolment hospital or clinic**		
Farafenni Hospital	721 (29%)	720 (29%)
Ngayen Sanjal	632 (26%)	632 (26%)
Nookunda	407 (16%)	408 (17%)
Kerewan	708 (29%)	708 (29%)
**Mother's education**		
No formal schooling	1617 (66%)	1655 (67%)
Less than primary	155 (6%)	167 (7%)
Completed primary, lower basic, or higher	521 (21%)	486 (20%)
Other*	170 (7%)	153 (6%)
**Source of drinking water**		
Improved water sources†	2308 (94%)	2317 (94%)
Household sanitation	..	..
Improved waste facilities†	1677 (68%)	1684 (68%)
**Oral rotavirus vaccine coverage (Rotarix)**		
None	34 (1%)	48 (2%)
One dose	14 (1%)	5 (<1%)
Two doses	2415 (98%)	2408 (98%)
Unknown	5 (<1%)	7 (<1%)

Data are n (%) or mean (SD).

* Including religious, Arabic, French, or other schools.

† UNICEF definition for improved water and sanitation.^27^

Serious adverse events occurred in 24 (1·0%) of 2474 participants in the vaccine group and 32 (1·3%) of 2462 in the placebo group; none were product related (table 2). Overall, adverse events were similar between groups (1098 [44·4%] of 2474 in the vaccine group *vs* 1096 [44·5%] of 2462 in the placebo group). In the reactogenicity cohort, severe (ie, grade 3) events were rare in both groups (two [1·1%] of 175 in the vaccine group *vs* one [0·6%] of 175 in the placebo group) and solicited adverse events was similar between groups (table 2). The proportion of solicited adverse events identified in the 7 days after each of three doses were similar between groups (appendix p 4). The safety cohort had similar frequency and severity of unsolicited adverse events between the groups, including for the most common events (diarrhoea, pyrexia, cough, respiratory infections, pneumonia, and vomiting). Vomiting within 30 min of dosing was infrequent without notable group differences (44 [1·8%] of 2474 *vs* 29 [1·2%] of 2462).

**Table 2:** Adverse events for reactogenicity and safety cohorts

			**Vaccine**	**Placebo**
Reactogenicity cohort			n=175	n=175
	Solicited adverse events by severity within 7 days of any dose			
		Mild (grade 1)	16/175 (9·1%)	24/175 (13·7%)
		Moderate (grade 2)	22/175 (12·6%)	23/175 (13·1%)
		Severe (grade 3)	2/175 (1·1%)	1/175 (0·6%)
	Solicited moderate and severe (grade ≥2) adverse events within 7 days of any dose			
		Diarrhoea or gastroenteritis	14/175 (8·0%)	14/175 (8·0%)
		Pyrexia	13/175 (7·4%)	11/175 (6·3%)
		Vomiting	1/175 (0·6%)	1/175 (0·6%)
		Urticaria	0/175	0/175
		Allergic dermatitis	1/175 (0·6%)	0/175
		Upper respiratory tract infection	0/175	2/175 (1·1%)
Safety cohort excluding reactogenicity cohort*			n=2299	n=2287
	Any adverse events by severity during trial			
		Mild (grade 1)	57/2299 (2·5%)	53/2287 (2·3%)
		Moderate (grade 2)	901/2299 (39·2%)	885/2287 (38·7%)
		Severe (grade 3)	19/2299 (0·8%)	18/2287 (0·8%)
		Life-threatening (grade 4)	0/2299	1/2287 (<0·1%)
		Deaths (grade 5)†	6/2299 (0·3%)	7/2287 (0·3%)
		Intervention-related grade ≥2 events	99/2299 (4·3%)	96/2287 (4·2%)
	Six most common moderate or severe (grade ≥2) adverse events during trial			
		Diarrhoea	409/2299 (17·8%)	423/2287 (18·5%)
		Pyrexia	292/2299 (12·7%)	295/2287 (12·9%)
		Cough	152/2299 (6·6%)	164/2287 (7·2%)
		Upper respiratory tract infection	82/2299 (3·6%)	69/2287 (3·0%)
		Pneumonia	82/2299 (3·6%)	68/2287 (3·0%)
		Vomiting	65/2299 (2·8%)	65/2287 (2·8%)
Safety cohort*			n=2474	n=2462
	One or more solicited or unsolicited adverse event		1098/2474 (44·4%)	1096/2462 (44·5%)
	Vomiting within 30 min of any dose		44/2474 (1·8%)	29/2462 (1·2%)
	Serious adverse events		24/2474 (1·0%)	32/2462 (1·3%)
	Intervention-related serious adverse events		0/2474	0/2462

A child could present with multiple adverse events (eg, vomiting and pyrexia), and each event is counted individually in the event-specific rows. However, when adverse events are presented by severity (ie, grade 1–5), each participant is counted once according to the highest severity grade.

* Any adverse event throughout the trial period.

† Two deaths in the vaccine group were identified in the reactogenicity cohort and were included here. No deaths were intervention related.

We estimated ETVAX coverage of circulating ETEC strains using all ETEC-positive strains detected in the ITT placebo group from enrolment (dose 1) to study end. Among 54 ETEC-positive MSD events, 49 included strains expressing ETVAX-targeted antigens, yielding an estimated vaccine coverage of 90·7% (appendix p 5). The remaining five vaccine-unrelated strains comprised heat-stable toxins only (three events), heat-stable toxin–CS14 (one event), and heat-stable toxin–CS14 plus heat-stable toxin only (one event).

Copathogens were common among ETEC-positive, vaccine-preventable outcomes. Of 39 ETEC-positive MSD events (13 [33%] vaccine and 26 [67%] placebo) in the per-protocol population, 37 (95%) had co-infections, of which 19 (51%) had one, 13 (35%) had two, and five (14%) had five copathogens. The most frequently detected copathogens were *Campylobacter* spp (19 [49%]), *Giardia lamblia* (16 [41%]), and enteroaggregative *E coli* (seven [18%]; appendix p 6).

In the primary efficacy analysis, which excluded ETEC-positive MSD events co-infected with *Cryptosporidium*, norovirus GII, rotavirus, or *Shigella* spp, vaccine efficacy was 26·6% (95% CI –58·2 to 66·0; p=0·43; table 3). In a secondary endpoint analysis in which no ETEC copathogens were excluded, efficacy increased to 48·2% (–0·9 to 73·4; p=0·053; NNV 164). With a definition of diarrhoea of three of more loose or liquid stools (rather than four or more) in 24 h, with no ETEC copathogens excluded, efficacy rose to 50·9% (9·4–73·4; p=0·023; NNV 125). When excluding events with *G lamblia* or *Cryptosporidium* spp, efficacy reached 80·6% (33·4–94·3; p=0·0092; NNV 161). ETVAX showed 21·4% efficacy against all-cause diarrhoea (2·0–36·9; p=0·032; NNV 50), with protection sustained throughout the study period (appendix p 8).

**Table 3:** Protective efficacy of ETVAX against ETEC-associated diarrhoea and all-cause diarrhoea in Gambian children in the per-protocol population, by endpoint

	**Vaccine (n=2041)**		**Placebo (n=2082)**		**Crude protective efficacy**	**Adjusted protective efficacy**		**Number needed to vaccinate to prevent one event**
	Events/child days of follow-up	Incidence per 100 000 follow-up days	Events/child days of follow-up	Incidence per 100 000 follow-up days	Estimate (95% CI)	Estimate (95% CI)	p value	
**Vaccine-preventable outcomes in which diarrhoea events presented with four or more loose or liquid stools in 24 h**								
Primary: ETEC diarrhoea excluding ETEC events co-infected with *Shigella* spp, rotavirus, norovirus GII, and *Cryptosporidium* spp	11/1 147 358	0·96	16/1 173 046	1·36	29·9 (−50·7 to 67·4)	26·6 (−58·2 to 66·0)*	0·43	NA
Secondary: ETEC diarrhoea regardless of copathogens	13/1 147 358	1·13	26/1 173 046	2·22	49·0 (1·0 to 73·7)	48·2 (−0·9 to 73·4)†	0·053	164
Exploratory: ETEC diarrhoea excluding ETEC events co-infected with enteric parasites (*Giardia lamblia* and *Cryptosporidium* spp)	3/1 147 358	0·26	16/1 173 046	1·36	80·9 (34·5 to 94·4)	80·6 (33·4 to 94·3)‡	0·0092	161
Secondary: diarrhoea regardless of cause	139/1 090 309	12·75	184/1 101 030	16·71	22·9 (4·8 to 37·6)	21·4 (2·0 to 36·9)§	0·032	50
**Vaccine-preventable outcomes in which diarrhoea events presented with three or more loose or liquid stools in 24 h**								
Secondary: ETEC diarrhoea excluding ETEC events co-infected with *Shigella* spp, rotavirus, norovirus GII, and *Cryptosporidium* spp	11/1 147 025	0·96	19/1 171 525	1·62	40·9 (−23·8 to 71·8)	38·8 (−28·7 to 70·9)¶	0·20	NA
Post hoc: ETEC diarrhoea regardless of copathogens	15/1 147 025	1·31	32/1 171 525	2·73	52·2 (12 to 74)	50·9 (9·4 to 73·4)‖	0·023	125

Adjusted protective efficacy from Cox proportional hazard model and crude vaccine efficacy from relative-risk estimates. The number needed to vaccinate is not calculated when adjusted p≥0·10. ETEC=enterotoxigenic *Escherichia coli*. GII=genogroup II. NA=not applicable.

* Efficacy was adjusted for enrolment centre and height-for-age Z score.

† Adjusted for enrolment centre.

‡ Adjusted for enrolment centre and age group.

§ Adjusted for enrolment centre and sex.

¶ Adjusted for enrolment centre and height-for-age Z score.

‖ Adjusted for enrolment centre and age group.

In a post-hoc subgroup analysis, efficacy among children receiving their first dose before age 9 months against ETEC-positive MSD irrespective of copathogens was 67·8% (95% CI 12·6 to 88·1; p=0·026; NNV 66) compared with 19·0% (–105·1 to 67·9; p=0·66) in those receiving the first dose at age 9–18 months (appendix p 7). For all-cause MSD, efficacy in the younger group was 24·8% (–1·8 to 44·4; p=0·066; NNV 33) versus 20·9% (–6·1 to 41·1; p=0·12) in the older group.

Efficacy estimates from the mITT analysis were consistent, but slightly lower for all per-protocol endpoints, except diarrhoea regardless of cause in the per-protocol population, for which the efficacy estimate was slightly higher, supporting the robustness of the findings (appendix p 9).

ETVAX induced antibody responses to multiple vaccine antigens. After three doses, participants in the vaccine group showed significantly higher GMT and GMFRs than participants in the placebo group after doses 2 and 3 for CFA/I IgA, LTB IgA, LTB IgG, and CS3 IgA (after dose 2 only; table 4). Responses to CS5 and CS6 were not significantly increased. LTB IgA responses peaked after two doses with a GMFR of 11·0 in the vaccine group versus 1·0 in the placebo group (p<0·0001). After the second dose, 55 (87%) of 63 participants for LTB-specific IgA and 56 (89%) for LTB-specific IgG had a GMFR of 2·0 or more (appendix p 10). Serum antibody responses remained consistent throughout the trial with no significant differences in GMFR between early and late enrolees for any vaccine antigen (all p≥0·39; appendix p 11).

**Table 4:** Geometric mean titres by dose, geometric mean fold rises from baseline at doses 2 and 3, and statistical comparison of fold rises between vaccine and placebo groups at doses 2 and 3, per response

	**Vaccine**			**Placebo**			**Geometric mean fold rise**					
	Before dosing (n=69)	After dose 2 (n=63)	After dose 3 (n=60)	Before dosing (n=53)	After dose 2 (n=46)	After dose 3 (n=46)	Vaccine after dose 2	p value	Vaccine after dose 3	p value	Placebo after dose 2	Placebo after dose 3
CFA/I IgA	35·6	45·8	106·3	26·2	22·8	43·8	1·4	0·0078	2·7	0·031	0·8	1·4
CS3 IgA	38·2	56·4	108·6	30·2	32·2	54·8	1·5	0·032	2·8	0·057	1·1	1·7
CS5 IgA	28·7	47·6	63·0	22·5	25·8	42·8	1·7	0·052	2·2	0·57	1·1	1·9
CS6 IgA	39·4	41·4	59·2	30·6	32·1	44·4	1·1	0·69	1·5	0·43	1·0	1·3
LTB IgA	100·2	1065·4	971·7	90·9	91·9	176·1	11·0*	<0·0001	9·4	<0·0001	1·0	1·7
LTB IgG	1140·5	10 293·4	14 148·5	1066·6	1183·4	2472·2	9·4*	<0·0001	11·2	<0·0001	1·0	2·2

Statistical comparison of geometric mean fold rise by Student's *t* test between vaccine and placebo after doses 2 and 3.

* Sample size n=47.

## Discussion

This phase 2b trial in The Gambia provides further evidence that ETVAX is safe and immunogenic. Although the primary endpoint was not met, secondary and exploratory analyses suggested that ETVAX protects against ETEC-positive MSD in infants and children, particularly when administered at younger ages and when children were not co-infected with enteroparasites at vaccination. Additional findings suggest protection against all-cause MSD, underscoring the vaccine's potential for broader diarrhoeal disease control. The following paragraphs explore these results and their implications.

ETVAX was well tolerated, with no increase in the frequency or severity of adverse events. Serious adverse events, the primary safety endpoint, were rare, occurred at similar rates between vaccine and placebo groups, and were unrelated to the vaccine. These findings align with previous ETVAX trials among Swedish, Zambian, Bangladeshi, and Finnish adults^28^, ^29^, ^30^, ^31^ as well as Bangladeshi and Zambian children.^11^, ^29^

A previous trial in Bangladesh^11^ that used the same vaccine dose as used in The Gambia found higher rates of mild and transient vomiting in children aged 6–23 months who received the vaccine compared with placebo. However, the Zambian trial showed no significant difference in vomiting rates for children aged 6–23 months with the same vaccine dose.^29^ In addition, in our trial, vomiting within 30 min of dosing was infrequent and similar in frequency between groups, and no increase in vomiting was observed during active home visits after each dose or among children seeking medical care after vaccination. Differences in the Bangladeshi and sub-Saharan populations could reflect variations in trial design, caregiver reporting, or population characteristics.

Most ETEC-positive MSD events involved enteric copathogens, and estimates of vaccine efficacy varied depending on their inclusion or exclusion. In the primary endpoint analysis that excluded participants who were co-infected with *Cryptosporidium*, norovirus GII, rotavirus, or *Shigella* spp, ETVAX efficacy was not significant. However, in a secondary analysis that included all ETEC-positive MSD events regardless of copathogens, ETVAX prevented nearly half of ETEC-positive diarrhoea events, approaching significance. In addition, when applying a more sensitive definition of diarrhoea (ie, three or more loose or liquid stools in 24 h *vs* four or more) while retaining the predefined signs and symptoms for dehydration, ETVAX prevented more than half of all ETEC-positive MSD events, reaching significance.

In an exploratory analysis, protection was highest (80·6%) and significant when ETEC-positive MSD events with enteroparasitic copathogens were excluded from analysis. Several mechanisms could explain this effect. When not excluded from analysis, parasitic copathogens might be the primary causative infection, leading to misclassification of disease aetiology and reducing efficacy estimates. Alternatively, enteroparasitic copathogens might suppress local immune responses or alter gut permeability, facilitating ETEC colonisation and toxin activity and compromising the host's ability to mount an effective defence.^32^, ^33^, ^34^ These mechanisms warrant investigation.

ETVAX also appeared to confer broader protection beyond ETEC-positive MSD. Similar to rotavirus vaccines, which prevent all-cause MSD by up to 27% in high child mortality settings,^35^ ETVAX reduced all-cause MSD by 21%. ETVAX might provide broader protection through several mechanisms. This effect might partly reflect protection against undetected ETEC due to the comparatively low sensitivity of culture-based diagnostics.^36^ The inclusion of dmLT and LCTBA antitoxin components might enhance innate and adaptive immune responses, contributing to non-specific protection,^37^ while cross-reactive antigens might offer partial protection against other enteric pathogens, particularly enterobacteria.^38^ Regardless of the mechanism, such broad protection has important public health implications, and results suggested that vaccinating 50 children with ETVAX could prevent one event of all-cause MSD.

Greater protection was observed against ETEC-positive MSD when vaccination began before age 9 months, reducing these events by two-thirds, whereas no significant reduction was observed among older children. These results might reflect enhanced development of naturally acquired immunity from the greater cumulative ETEC exposure over time in older children. Contrastingly, although ETVAX showed protection against all-cause diarrhoea overall, no age-related effect was detected (p=0·06) in children vaccinated before age 9 months, potentially due to reduced statistical power from smaller sample sizes after age stratification.

A major challenge in the development of an ETEC vaccine is the heterogeneity of clinical ETEC strains due to toxin and colonisation factor diversity. ETVAX antigens matched 90·7% of local circulating ETEC strains. A study in south Asia (Bangladesh) supported that ETVAX-associated colonisation factors are the most prevalent in those settings.^39^ Still, coverage might vary in other ETEC-endemic regions depending on the proportion of circulating strains expressing the vaccine-targeted antigens.

In addition to the substantial coverage of circulating ETEC strains in this trial, ETVAX elicited good serum immune responses against CFA/I, CS3, and LTB antigens, with markedly increased LTB responses observed after two doses. There were diminished responses to CS5 and CS6; however, the immunogenicity cohort was not primarily dimensioned to evaluate responses to individual colonisation factors. Rather, it aimed to establish whether vaccine-induced immune responses remained consistent from the start and at the end of enrolment. The results indicated no decline in vaccine immunogenicity across early and late enrolment, supporting vaccine stability over time.

Our trial had limitations. Identifying the causative pathogen in polymicrobial diarrhoea remains challenging. In our primary analysis, we excluded ETEC-positive events co-infected with *Cryptosporidium* spp, norovirus GII, rotavirus, or *Shigella* spp given that their inclusion in the efficacy analysis might mask ETEC-specific efficacy. However, excluding these events led to an underestimation of ETVAX efficacy. Including all ETEC-positive events, regardless of copathogens, provided an increased and significant efficacy estimate and suggested protection against the previously excluded events. In this trial, the stool count for diarrhoea was revised after 1 trial year from four or more to three or more loose or liquid stools in 24 h to align with widely used clinical definitions. Despite potential undercount of events before the change to three or more stools, efficacy estimates for ETEC regardless of copathogens were consistent across the two definitions (48·2% *vs* 50·9%) but with greater statistical precision for the revised count (p=0·053 *vs* p=0·023) due to increased incidence. Future trials should use ≥3 stools as the case definition while using similar requirements for MSD. Although ETVAX is designed to elicit protection via antibodies against colonisation factors and heat-labile toxins, three ETVAX strains were O78 *E coli*, a common ETEC O antigen.^40^ We did not assess immune responses to O78 LPS antigen nor serotype ETEC isolates. A possibility is that part of the observed ETEC protection might be due to vaccine-enhanced responses against this O78 antigen. We did not collect serum samples after the first dose because we were compelled to reduce the number of blood samples collected. This situation limited our ability to determine peak anti-heat-labile toxins responses after the first dose. ETVAX appeared to reduce the incidence of all-cause diarrhoea in this trial. However, because we only tested for ETEC and ETEC copathogens in diarrhoea events, we could not assess whether the vaccine independently protected against other diarrhoea pathogens such as *Shigella* spp, *Campylobacter* spp, or different enteric *E coli* pathotypes. The broader protection observed might reflect unmeasured ETVAX effects against these organisms. In this analysis, probability values were unadjusted for multiple comparisons, and results should be viewed as hypothesis generating in anticipation of planning for a phase 3 trial. Finally, high co-infection rates and culture-based ETEC diagnostics can misclassify case aetiology, although prespecified consequences of copathogens were considered analytically.

Among ETEC vaccine candidates, ETVAX is the most clinically advanced, having completed a phase 2b efficacy trial among Finnish travellers to Benin^31^ and now a large paediatric trial in The Gambia. Other candidates in clinical development include CVD1208S-122, a live attenuated candidate expressing ETEC antigens (NCT04634513). ShigETEC, a live oral combination vaccine targeting both *Shigella* and ETEC, is undergoing phase 2b evaluation in a controlled human infection model that uses a *Shigella flexneri* 2a to assess vaccine efficacy, but no ETEC challenge (NCT07049159). Additional candidates include adhesin-based subunit vaccines that target the adhesive tip protein of bacterial fimbriae to block pathogen attachment to host cells, including the CFA/I tip adhesin, for which a human challenge trial has been completed,^41^ and CssBA (CS6), which is being evaluated (NCT06692907).

One of the subsequent priorities for deploying ETVAX is to conduct feasibility studies to identify models for integrating its dosing schedule into routine service delivery platforms. The safety and efficacy of ETVAX in the youngest age group suggests the potential to follow the pentavalent vaccine schedule or to give ETVAX at the same time as measles-containing, yellow fever, meningococcal A, measles–mumps–rubella, or measles–rubella vaccines, and at the same time as vitamin supplementation campaigns or other periodic campaigns, such as deworming. Alternative dosing models, such as modifying the current one-day, 15-day, and 90-day regimen to once a month or initiating the first of three doses before age 6 months could improve alignment and facilitate integration with existing platforms. If coadministration with existing childhood vaccines is pursued, dedicated trials assessing safety and immunogenicity will be essential.

Another required step will be a pivotal phase 3 efficacy trial. This trial would confirm protective efficacy and estimate vaccine coverage for circulating ETEC strains in diverse settings. The next trial will focus on enrolling children aged 6–9 months with the primary endpoint being culture-confirmed ETEC-positive events, excluding enteroparasitic copathogens. Efforts will also focus on establishing commercial manufacturing and engaging with global health partners (eg, WHO and Gavi, the Vaccine Alliance) and Ministries of Health for considering ETVAX introduction.

In conclusion, ETVAX was safe, immunogenic, and showed protection against ETEC-positive MSD in the presence of copathogens. The vaccine also reduced all-cause MSD. Greater efficacy was observed when vaccination occurred before age 9 months, supporting early administration in endemic settings to maximise protection. Protection was further enhanced in children who were not infected with enteroparasites at the time of vaccination. These findings support advancing ETVAX to a pivotal phase 3 trial.

### Contributors

### Data sharing

The protocol, statistical analysis plan, and de-identified data underlying the reported results are available upon reasonable request from Scandinavian Biopharma (info@scandinavianbiopharma.se) beginning 3 months after and ending 3 years after publication.

## Declaration of interests

NC and BSj are employees and minority shareholders of Scandinavian Biopharma, which holds some commercial rights to the vaccine tested in this study. A-MS is a shareholder of Gotovax, which might receive a small royalty on sales of the ETEC vaccine if it becomes a commercial product. NC and A-MS have patents PCT/EP2012/067598-PCT and PCT/EP2011/065784-PCT. All other authors declare no competing interests.

## References

[1] WHO Enterotoxigenic *Escherichia coli*. https://www.who.int/teams/immunization-vaccines-and-biologicals/diseases/enterotoxigenic-escherichia-coli-(etec).

[2] Colston JM, Zaitchik BF, Badr HS (2022). Associations between eight earth observation-derived climate variables and enteropathogen infection: an independent participant data meta-analysis of surveillance studies with broad spectrum nucleic acid diagnostics. Geohealth.

[3] Khalil I, Walker R, Porter CK (2021). Enterotoxigenic *Escherichia coli* (ETEC) vaccines: priority activities to enable product development, licensure, and global access. Vaccine.

[4] Guerrant RL, Oriá RB, Moore SR, Oriá MO, Lima AA (2008). Malnutrition as an enteric infectious disease with long-term effects on child development. Nutr Rev.

[5] Vidal RM, Muhsen K, Tennant SM (2019). Colonization factors among enterotoxigenic *Escherichia coli* isolates from children with moderate-to-severe diarrhea and from matched controls in the Global Enteric Multicenter Study (GEMS). PLoS Negl Trop Dis.

[6] Fleckenstein JM, Hardwidge PR, Munson GP, Rasko DA, Sommerfelt H, Steinsland H (2010). Molecular mechanisms of enterotoxigenic *Escherichia coli* infection. Microbes Infect.

[7] WHO (June 18, 2020). PDVAC Executive Summary 2020: update on development of enterotoxigenic *E. coli* (ETEC) vaccines. https://www.who.int/publications/m/item/pdvac-executive-summary-2020-update-on-development-of-etec-vaccines.

[8] Holmgren J, Bourgeois L, Carlin N (2013). Development and preclinical evaluation of safety and immunogenicity of an oral ETEC vaccine containing inactivated *E. coli* bacteria overexpressing colonization factors CFA/I, CS3, CS5 and CS6 combined with a hybrid LT/CT B subunit antigen, administered alone and together with dmLT adjuvant. Vaccine.

[9] Lebens M, Shahabi V, Bäckström M, Houze T, Lindblad N, Holmgren J (1996). Synthesis of hybrid molecules between heat-labile enterotoxin and cholera toxin B subunits: potential for use in a broad-spectrum vaccine. Infect Immun.

[10] Clements JD, Norton EB (2018). The mucosal vaccine adjuvant LT(R192G/L211A) or dmLT. MSphere.

[11] Qadri F, Akhtar M, Bhuiyan TR (2020). Safety and immunogenicity of the oral, inactivated, enterotoxigenic *Escherichia coli* vaccine ETVAX in Bangladeshi children and infants: a double-blind, randomised, placebo-controlled phase 1/2 trial. Lancet Infect Dis.

[12] National Institute of Allergy and Infectious Diseases Division of AIDS (DAIDS) (July, 2017). Table for grading the severity of adult and pediatric adverse events, version 2.1. https://rsc.niaid.nih.gov/sites/default/files/daidsgradingcorrectedv21.pdf.

[13] Uppsala Monitoring Centre, WHO Collaborating Centre (June 5, 2013). The use of the WHO–UMC system for standardised case causality assessment. https://www.who.int/docs/default-source/medicines/pharmacovigilance/whocausality-assessment.pdf.

[14] WHO (March 7, 2024). Diarrhoeal disease. https://www.who.int/news-room/fact-sheets/detail/diarrhoeal-disease.

[15] WHO, Department of Child and Adolescent Health and Development (CAH), UNICEF (2005).

[16] Ruuska T, Vesikari T (1990). Rotavirus disease in Finnish children: use of numerical scores for clinical severity of diarrhoeal episodes. Scand J Infect Dis.

[17] Roy C, Robert D, Bénéjat L (2020). Performance evaluation of the Novodiag Bacterial GE+ multiplex PCR assay. J Clin Microbiol.

[18] Sjöling A, Wiklund G, Savarino SJ, Cohen DI, Svennerholm AM (2007). Comparative analyses of phenotypic and genotypic methods for detection of enterotoxigenic *Escherichia coli* toxins and colonization factors. J Clin Microbiol.

[19] Lopez-Vidal Y, Svennerholm AM (1990). Monoclonal antibodies against the different subcomponents of colonization factor antigen II of enterotoxigenic *Escherichia coli*. J Clin Microbiol.

[20] Viboud GI, Binsztein N, Svennerholm AM (1993). Characterization of monoclonal antibodies against putative colonization factors of enterotoxigenic *Escherichia coli* and their use in an epidemiological study. J Clin Microbiol.

[21] Kotloff KL, Nataro JP, Blackwelder WC (2013). Burden and aetiology of diarrhoeal disease in infants and young children in developing countries (the Global Enteric Multicenter Study, GEMS): a prospective, case-control study. Lancet.

[22] Breurec S, Vanel N, Bata P (2016). Etiology and epidemiology of diarrhea in hospitalized children from low income country: a matched case-control study in Central African Republic. PLoS Negl Trop Dis.

[23] Iturriza-Gómara M, Jere KC, Hungerford D (2019). Etiology of diarrhea among hospitalized children in Blantyre, Malawi, following rotavirus vaccine introduction: a case-control study. J Infect Dis.

[24] Mero S, Timonen S, Lääveri T (2021). Prevalence of diarrhoeal pathogens among children under five years of age with and without diarrhoea in Guinea-Bissau. PLoS Negl Trop Dis.

[25] Pelkonen T, Dos Santos MD, Roine I (2018). Potential diarrheal pathogens common also in healthy children in Angola. Pediatr Infect Dis J.

[26] Lin DY, Wei LJ, Ying Z (1993). Checking the Cox model with cumulative sums of Martingale-based residuals. Biometrika.

[27] UNICEF (Feb 22, 2018). Progress on drinking-water, sanitation, and hygiene: 2017 update and DSG baselines. https://www.who.int/publications/i/item/9789241512893.

[28] Lundgren A, Bourgeois L, Carlin N (2014). Safety and immunogenicity of an improved oral inactivated multivalent enterotoxigenic *Escherichia coli* (ETEC) vaccine administered alone and together with dmLT adjuvant in a double-blind, randomized, placebo-controlled phase I study. Vaccine.

[29] Sukwa N, Mubanga C, Hatyoka LM (2023). Safety, tolerability, and immunogenicity of an oral inactivated ETEC vaccine (ETVAX) with dmLT adjuvant in healthy adults and children in Zambia: an age descending randomised, placebo-controlled trial. Vaccine.

[30] Akhtar M, Chowdhury MI, Bhuiyan TR (2019). Evaluation of the safety and immunogenicity of the oral inactivated multivalent enterotoxigenic *Escherichia coli* vaccine ETVAX in Bangladeshi adults in a double-blind, randomized, placebo-controlled phase I trial using electrochemiluminescence and ELISA assays for immunogenicity analyses. Vaccine.

[31] Kantele A, Riekkinen M, Jokiranta TS (2023). Safety and immunogenicity of ETVAX, an oral inactivated vaccine against enterotoxigenic *Escherichia coli* diarrhoea: a double-blinded, randomized, placebo-controlled trial amongst Finnish travellers to Benin, West Africa. J Travel Med.

[32] Akoolo L, Rocha SC, Parveen N (2022). Protozoan co-infections and parasite influence on the efficacy of vaccines against bacterial and viral pathogens. Front Microbiol.

[33] Klimczak S, Packi K, Rudek A (2024). The influence of the protozoan *Giardia lamblia* on the modulation of the immune system and alterations in host glucose and lipid metabolism. Int J Mol Sci.

[34] Wait LF, Dobson AP, Graham AL (2020). Do parasite infections interfere with immunisation? A review and meta-analysis. Vaccine.

[35] Bergman H, Henschke N, Hungerford D (2021). Vaccines for preventing rotavirus diarrhoea: vaccines in use. Cochrane Database Syst Rev.

[36] Miller JM, Binnicker MJ, Campbell S (2018). A guide to utilization of the microbiology laboratory for diagnosis of infectious diseases: 2018 update by the Infectious Diseases Society of America and the American Society for Microbiology. Clin Infect Dis.

[37] Akhtar M, Nizam NN, Basher SR (2021). dmLT adjuvant enhances cytokine responses to T cell stimuli, whole cell vaccine antigens and lipopolysaccharide in both adults and infants. Front Immunol.

[38] Terrinoni M, Holmgren J, Turbyfill KR, Van De Verg L, Maier N, Walker R (2025). Potential for a combined oral inactivated whole-cell vaccine against ETEC and *Shigella*: preclinical studies supporting feasibility. Vaccines.

[39] Akhtar M, Begum YA, Isfat Ara Rahman S (2024). Age-dependent pathogenic profiles of enterotoxigenic *Escherichia coli* diarrhea in Bangladesh. Front Public Health.

[40] Qadri F, Svennerholm AM, Faruque AS, Sack RB (2005). Enterotoxigenic *Escherichia coli* in developing countries: epidemiology, microbiology, clinical features, treatment, and prevention. Clin Microbiol Rev.

[41] Gutiérrez RL, Porter CK, Harro C (2024). Efficacy evaluation of an intradermally delivered enterotoxigenic *Escherichia coli* CF antigen I fimbrial tip adhesin vaccine coadministered with heat-labile enterotoxin with LT(R192G) against experimental challenge with enterotoxigenic *E coli* H10407 in healthy adult volunteers. Microorganisms.

