# Supplementary material for: Efficacy of ETVAX, a vaccine against enterotoxigenic Escherichia coli-positive diarrhoea in Gambian children: a double-blind, randomised, placebo-controlled, phase 2b trial
Source: Lancet Infect Dis. 2026 Jun;26(6):627–37. doi: 10.1016/S1473-3099(25)00774-1 (PMC13241577; doi:10.1016/S1473-3099(25)00774-1)
Supplement: Supplementary appendix [file mmc1.pdf]

THE LANCET
Infectious Diseases

Supplementary appendix

This appendix formed part of the original submission and has been peer reviewed. We post it as supplied by the authors.

Supplement to: Hossain MJ, Secka F, Sanyang LC, et al. Efficacy of ETVAX, a vaccine against enterotoxigenic *Escherichia coli*-positive diarrhoea in Gambian children: a double-blind, randomised, placebo-controlled, phase 2b trial. *Lancet Infect Dis* 2026; published online Feb 16. https://doi.org/10.1016/S1473-3099(25)00774-1.

Appendix

Table of Contents

Laboratory Diagnostics ... 2

Table S1: Solicited Adverse Events (Grade 2 or 3) Within 7 Days of Each ETVAX Dose, Reactogenicity Cohort ... 4

Table S2: Frequency of single and mixed vaccine related and vaccine unrelated moderate-to-severe diarrhoea ETEC case strains in placebo group from enrolment (dose 1) to end of trial ... 5

Table S3: Frequency of Enteric Copathogens Among ETEC-positive Moderate-to-Severe Diarrhoea by Treatment Group ... 6

Table S4: Protective Efficacy of ETVAX against ETEC-positive Diarrhoea and All-cause Diarrhoea in Gambian Children by Age (6 to <9 months vs. 9 to 18 month) at first dose, Per-Protocol Population, Post-hoc Analysis ... 7

Figure F1: Cumulative Incidence Curve for Time in Days from Seven Days after Third Dose to First Episode of Moderate-to-Severe Diarrhoea Regardless of Aetiology for ETVAX and Placebo Recipients ... 8

Table S5: Protective Efficacy of ETVAX against ETEC-positive Moderate-to-Severe Diarrhoea and Moderate-to-Severe Diarrhoea Regardless of Aetiology in Gambian Children, modified Intention-to-treat population ... 9

Table S6: Percent of children with geometric mean fold rise ≥2 and ≥4 for serum IgA antibody responses to CFA/I, CS3, CS5, CS6, and LTB and IgG responses to LTB after 2 and 3 doses of ETVAX or placebo ... 10

Table S7: Geometric Mean Fold Raise (GMFR) of serum antibody responses to ETVAX antigens in ETVAX recipients at the beginning (≤ June 2021) and end of the enrolment (≥ February 2022) ... 11

Laboratory Diagnostics

Stool and rectal swabs were collected from MSD cases. Rectal swabs (FLOQ Swab, Copan Diagnostics, Italy) were placed in eNAT or mNAT S Medium tubes (Hologic, USA), and stool samples were collected in sterile, 50 mL polystyrene containers with metal caps and printed labels. If a stool sample was not produced during the clinic visit, study staff followed up with the caregiver within 24 hours by home visit or during a child's return clinic visit.

Rectal swab specimens were transported in cold boxes (2–8°C) to one of three local laboratories (Farafenni, Ngayen Sangal, Kerewan) and immediately tested for ETEC toxin genes (LT, STh, STp) using the Novodiag Bacterial GE molecular assay (Hologic, USA)[1]. If ETEC was not detected, specimens were discarded. If ETEC was detected, the corresponding whole stool sample, along with stool-inoculated Buffered Glycerol Saline, Cary-Blair, and eNAT transport media, was transported in a cold box (2–8 °C) to the MRCG Keneba laboratory. Upon arrival, the whole stool and stool-inoculated eNATs or mNAT were immediately stored at −70°C, while the Buffered Glycerol Saline and Cary-Blair samples were cultured for the isolation of *E coli*, *Shigella* spp, *Salmonella* spp, and *Vibrio cholerae* using standard microbiological techniques.

From each ETEC-positive culture, ten individual *E coli*-like colonies were selected, each placed in a 15% glycerol cryovial, and transported by cold box (2–8°C) to the MRCG, Fajara laboratory and stored at -70°C until tested. A pool of the 10 colonies was screened by PCR for LT, STh (human), and STp (porcine) genes developed at the University of Gothenburg[2] using the QIAxcel Advanced System (QIAGEN, Germany). If the pooled sample was positive, each colony was individually tested using the same assay. Individual colonies positive for ETEC were further characterised for CFs by dot blot assay, developed at the University of Gothenburg[2], at MRCG, Fajara. Confirmed ETEC-positive isolates were shipped on dry ice (−90°C to −20°C) in temperature-controlled shipping containers via World Courier to the University of Gothenburg for quality control analyses of CFs by dot blot and toxin expression by GM1 ELISA[2-4].

ETEC culture-positive stool samples inoculated in eNat preservative medium were also transported in dry ice using temperature-controlled shipping containers (−90°C to −20°C) via World Courier to Synlab (Helsinki, Finland) for multiplex PCR screening (Amplidiag, Hologic, USA) for other enteric viral, bacterial, and parasitic pathogens. *Campylobacter* spp were detected by antigen ELISA (Premier Campy, Meridian Bioscience, USA) at MRCG, Fajara. Quality control for ETEC toxin and CF expression was conducted at the University of Gothenburg.

References

1. Roy C, Robert D, Benejat L, et al. Performance Evaluation of the Novodiag Bacterial GE+ Multiplex PCR Assay. *J Clin Microbiol* 2020; **58**(10).

2. Sjoling A, Wiklund G, Savarino SJ, Cohen DI, Svennerholm AM. Comparative analyses of phenotypic and genotypic methods for detection of enterotoxigenic Escherichia coli toxins and colonization factors. *J Clin Microbiol* 2007; **45**(10): 3295-301.

3. Lopez-Vidal Y, Svennerholm AM. Monoclonal antibodies against the different subcomponents of colonization factor antigen II of enterotoxigenic Escherichia coli. *J Clin Microbiol* 1990; **28**(9): 1906-12.

4. Viboud GI, Binsztein N, Svennerholm AM. Characterization of monoclonal antibodies against putative colonization factors of enterotoxigenic Escherichia coli and their use in an epidemiological study. *J Clin Microbiol* 1993; **31**(3): 558-64.

Table S1: Solicited Adverse Events (Grade 2 or 3) Within 7 Days of Each ETVAX Dose, Reactogenicity Cohort

	Dose 1 (day 1)		Dose 2 (day 15)		Dose 3 (day 90)	
**Solicited Adverse Event**	**Vaccine (n=175)**	**Placebo (n=175)**	**Vaccine (n=155)**	**Placebo (n=164)**	**Vaccine (n=156)**	**Placebo (n=150)**
Diarrhoea/Gastroenteritis	5 (2·9%)	3 (1·7%)	4 (2·6%)	4 (2·4%)	6 (3·9%)	6 (4·0%)
Pyrexia	7 (4·0%)	3 (1·7%)	2 (1·3%)	4 (2·4%)	5 (3·2%)	4 (2·7%)
Vomiting	0 (0·0%)	0 (0·0%)	1 (0·7%)	1 (0·6%)	0 (0·0%)	0 (0·0%)
Urticaria	0 (0·0%)	0 (0·0%)	0 (0·0%)	0 (0·0%)	0 (0·0%)	0 (0·0%)
Allergic Dermatitis	0 (0·0%)	0 (0·0%)	1 (0·7%)	0 (0·0%)	0 (0·0%)	0 (0·0%)
Upper Respiratory Tract Infection	0 (0·0%)	1 (0·6%)	0 (0·0%)	1 (0·6%)	0 (0·0%)	0 (0·0%)
Any	11 (6·3%)	7 (4·0%)	6 (3·9%)	10 (6·1%)	9 (5·8%)	9 (6·0%)

Note: A child may present with more than one grade 2 or 3 adverse events (eg, diarrhoea and pyrexia) and each event is counted separately; for “Any” solicited adverse event, each child is counted only once.

Table S2: Frequency of single and mixed vaccine related and vaccine unrelated moderate-to-severe diarrhoea ETEC case strains in placebo group from enrolment (dose 1) to end of trial

ETEC strains-CFs	n (n=54)
**Vaccine Related Strains (n=49, 90·7%)**	
**Single ETEC strains**	
LT only*	10
LTST only	8
LTST-CS3	3
LTST-CS5&CS6	3
LTST-CS12	3
LTST-CS14	2
ST-CFA/I	2
ST-CS6	2
LTST-CS2&CS3	1
**Mixed ETEC strains†**	
ST-CFA/I, ST only	5
ST-CS6, ST only	2
LTST-CS6, LTST-CS5&6, LTST only	1
LTST-CS5&6, LTST only	1
LT-CS6, LT-CS5&6, LT only	1
LT-CS6, LT-only	1
LTST-CS14, ST-CS14, ST-only	1
LTST-CS14, LT-CS14, ST-CS14	1
LTST-CS14, ST only	1
LTST-CS3, LTST only	1
**Vaccine Unrelated Strains**	
ST only	3
ST-CS14, ST only	1
ST-CS14	1

*"LT only, ST only, and LTST only" are strains without colonization factors †At least one strain must express a ETVAX vaccine antigen (ie, LT, LTST, CFA/1, CS3, C5 or CS6)

Table S3: Frequency of Enteric Copathogens Among ETEC-positive Moderate-to-Severe Diarrhoea by Treatment Group, Per Protocol Population

**Copathogens**	**Vaccine (n=13)***	**Placebo (n=26)***	**Total (n=39)***
Campylobacter spp.	5	14	19
Giardia lamblia	9	7	16
Enteroaggregative *E. coli* (EAEC)	4	3	7
Enteropathogenic *E. coli* (EPEC)	1	4	5
Norovirus GII	0	5	5
Astrovirus	1	2	3
Cryptosporidium spp.	1	2	3
Shigella spp.	0	3	3
Dientamoeba fragilis	2	1	3
Rotavirus	1	2	3
Adenovirus	2	0	2
Sapovirus	1	1	2
Norovirus GI	0	1	1

* ETEC-positive cases

Table S4: Protective Efficacy of ETVAX against ETEC-positive Diarrhoea and All-cause Diarrhoea in Gambian Children by Age (6 to <9 months vs. 9 to 18 month) at first dose, Per-Protocol Population, Post-hoc Analysis

Age (months) at first dose	Vaccine (n=2041)		Placebo (n=2082)		Relative Risk (95% CI)	Vaccine Efficacy		Number needed to vaccinate to prevent one case*
	Cases/ follow-up days	Incidence/ 100,000 days	Cases/ follow-up days	Incidence/ 100,000 days		Estimate (95% CI)	p-value	
**Moderate-to-severe ETEC-associated diarrhoea regardless of copathogens**								
6 to <9	5/372,605	1·34	16/386,714	4·14	0·322 (0·119 to 0·874)	67·8 (12·6 to 88·1)	0·026	67
**9 to 18**	8/774,210	1·03	10/786,241	1·27	0·812 (0·321 to 2.051)	18·8 (-105·1 to 67·9)	0·66	NA
**Moderate-to-severe diarrhoea regardless of cause**								
6 to <9	65/345,733	18·80	89/358,788	24·81	0·752 (0·556 to 1·018)	24·8 (-1·80 to 44·4)	0·065	33
9 to 18	74/744,576	9·94	95/742,242	12·80	0·791 (0·589 to 1·062)	20·9 (-6·1 to 41·1)	0·12	NA

* The number needed to vaccinate is not calculated when the p-values are ≥0·10.

Figure F1: Cumulative Incidence Curve for Time in Days from Seven Days after Third Dose to First Episode of Moderate-to-Severe Diarrhoea Regardless of Aetiology for ETVAX and Placebo Recipients

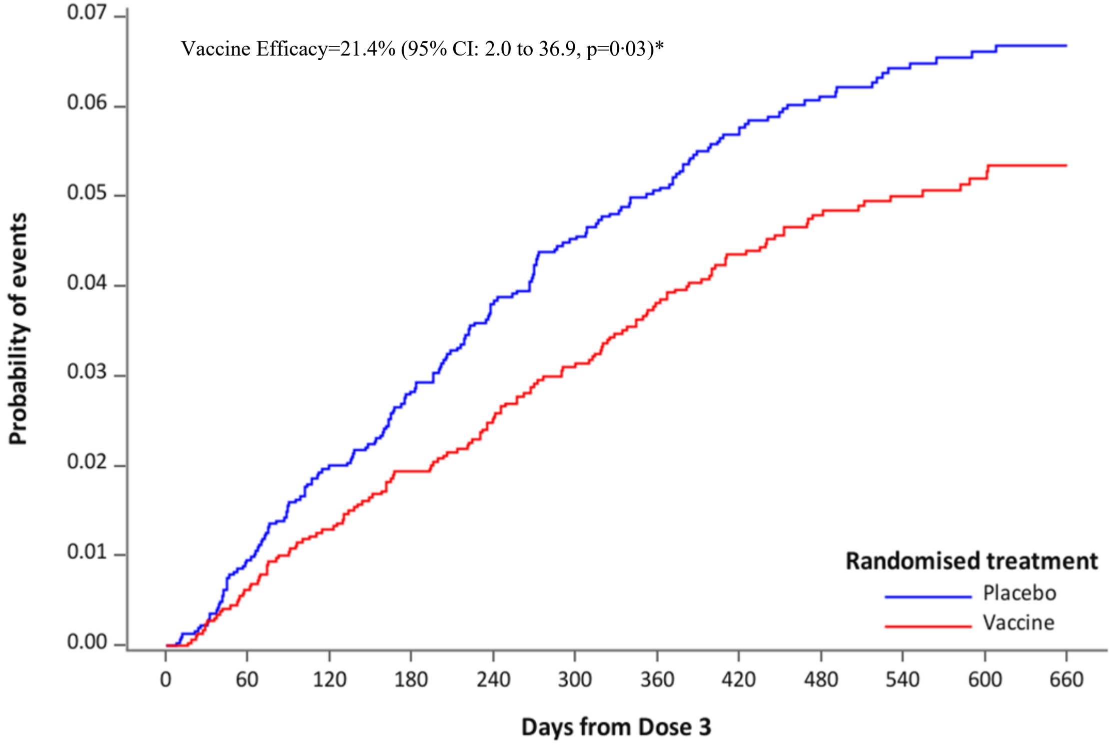

No. at Risk												
Placebo	2040	2008	1977	1951	1921	1901	1886	1769	1516	1355	1074	997
Vaccine	1987	1966	1947	1928	1911	1891	1872	1792	1536	1346	1066	998
**No. of events**												
Placebo	0	29	60	84	113	132	147	166	174	180	183	184
Vaccine	0	18	37	55	72	88	106	120	130	134	137	139

Note Number of observations censored before third dose: placebo 42, vaccine 54.
*Results generated from Cox's model adjusting for enrolment centre and sex

Table S5: Protective Efficacy of ETVAX against ETEC-associated Diarrhoea and All Cause Diarrhoea in Gambian Children, Per-Protocol Population, Modified Intention-to-treat Population

					Protective Efficacy			
	Vaccine (n=2184)		Placebo (n=2177)		Crude	Adjusted		
Endpoint	Cases/child days of follow-up	Incidence per 100,000 days of follow-up	Cases/child days of follow-up	Incidence per 100,000 days of follow-up	Estimate (95% CI)	Estimate (95% CI)	p-value	Number needed to vaccinate to prevent one case
**Vaccine Preventable Outcomes where diarrhoea cases presented with 4 or more loose or liquid stools in 24 hours**								
Primary: ETEC diarrhoea excluding ETEC cases coinfected with *Shigella*, rotavirus, norovirus GII, and *Cryptosporidium*	13/1,227,480	1·06	18/1,225,030	1·47	28 (-46·6 to 64·6)	26·1 (-50·9 to 63·8)*	0·41	Not Applicable
Secondary: ETEC diarrhoea regardless of copathogens	17/1,227,480	1·38	29/1,225,030	2·37	41·6 (-6 to 67·8)	40·6 (-8·1 to 67·4)†	0·088	181
Exploratory: ETEC diarrhoea excluding ETEC cases coinfected with enteric parasites (Giardia lamblia and Cryptosporidium)	7/1,227,480	0·57	18/1,225,030	1·47	61·2 (7·4 to 83·8)	60·7 (5·9 to 83·6)‡	0·036	198
Secondary: Diarrhoea regardless of aetiology	150/1,161,717	12·91	195/1,145,897	17·02	23·3 (5·9 to 37·5)	22·9 (4·6 to 37·7)¶	0·017	48
**Vaccine Preventable Outcomes where diarrhoea cases presented with 3 or more loose or liquid stools in 24 hours**								
Secondary: ETEC diarrhoea excluding ETEC cases coinfected with *Shigella*, rotavirus, norovirus GII, and *Cryptosporidium*	15/1,225,895	1·22	21/1,223,167	1·72	28·8 (-37·7 to 63·2)	27·7 (-40·4 to 62·7)‖	0·34	Not Applicable
Post hoc: ETEC diarrhoea regardless of ETEC copathogens	22/1,225,895	1·79	36/1,223,167	2·94	39·1 (-3·2 to 64)	38·1 (-5·3 to 63·6)**	0·077	155

Note: Adjusted protective efficacy from Cox proportional hazard model and crude Protective Efficacy VE from relative risk estimates the number needed to vaccinate is not calculated when adjusted p-values are ≥0·10.

* The efficacy was adjusted for enrolment centre and height-for-age z-score; † Adjusted for enrolment centre, age group (below/above 12 months old) and height-for-age z-score; ‡ Adjusted for enrolment centre and age group (below/above 12 months old); § Adjusted for enrolment centre; ¶ Adjusted for enrolment centre and gender; ‖ Adjusted for enrolment centre and height-for-age z-score; ** Adjusted enrolment centre and age group (below/above 12 months old)

Table S6: Percent of children with geometric mean fold rise ≥2 and ≥4 for serum IgA antibody responses to CFA/I, CS3, CS5, CS6, and LTB and IgG responses to LTB after 2 and 3 doses of ETVAX or placebo

Antibody Response	Dose 2				Dose 3			
	Vaccine (n = 63)		Placebo (n = 46)		Vaccine (n = 60)		Placebo (n = 45)	
	≥2-fold (n)	≥4-fold (n)	≥2-fold (n)	≥4-fold (n)	≥2-fold (n)	≥4-fold (n)	≥2-fold (n)	≥4-fold (n)
CFA/I IgA	22% (14)	13% (8)	4% (2)	2% (1)	48% (29)	28% (17)	29% (13)	18% (8)
CS3 IgA	33% (21)	17% (11)	11% (5)	2% (1)	52% (31)	30% (18)	40% (18)	17% (8)
CS5 IgA	29% (18)	17% (11)	20% (9)	7% (3)	47% (28)	27% (16)	49% (22)	24% (11)
CS6 IgA	21% (13)	3% (2)	15% (7)	2% (1)	45% (27)	20% (12)	38% (17)	11% (5)
LTB IgA	87% (55)	79% (50)	21% (10)	11% (5)	83% (50)	72% (43)	38% (17)	24% (11)
LTB IgG	89% (56)	76% (48)	13% (6)	4% (2)	83% (50)	73% (44)	44% (20)	27% (12)

Table S7: Geometric Mean Fold Raise (GMFR) of serum antibody responses to ETVAX antigens in ETVAX recipients at the beginning (≤ June 2021) and end of the enrolment (≥ February 2022)

	Post Dose 2		Post Dose 3	
**Antibody**	**GMFR ≤ June 2021 (n=34)**	**GMFR ≥February 2022 (n=29)**	**GMFR ≤ June 2021 (n=34)**	**GMFR ≥ February 2022 (n=26)**
CFA/I IgA	1·3	1·5	3·3	2·2
CS3 IgA	1·4	1·7	2·7	2·9
CS5 IgA	1·6	1·8	1·9	2·5
CS6 IgA	1·0	1·1	1·4	1·7
LTB IgA	12·7	9·3	10·3	8·3
LTB IgG	10·3	8·3	12·0	10·3

Note: No statistically significant differences by Students T-test were detected between time points (beginning vs. end of enrolment) for either dose; All p-values were ≥0·39.
